# Circulatory metabolites trigger ex vivo arterial endothelial cell dysfunction in population chronically exposed to diesel exhaust

**DOI:** 10.1186/s12989-022-00463-0

**Published:** 2022-03-22

**Authors:** Wenting Cheng, Huanhuan Pang, Matthew J. Campen, Jianzhong Zhang, Yanting Li, Jinling Gao, Dunqiang Ren, Xiaoya Ji, Nathaniel Rothman, Qing Lan, Yuxin Zheng, Shuguang Leng, Zeping Hu, Jinglong Tang

**Affiliations:** 1 Department of Occupational and Environmental Health, School of Public Health, Qingdao University, Qingdao, 266021 Shandong China; 2 School of Pharmaceutical Sciences, Tsinghua University, Beijing, 100084 China; 3 Department of Pharmaceutical Sciences, College of Pharmacy, University of New Mexico, Albuquerque, NM 87131 USA; 4 Department of Respiratory Medicine, Affiliated Hospital of Medical College of Qingdao University, Qingdao University, Qingdao, 266021 Shandong China; 5 Division of Cancer Epidemiology and Genetics, National Cancer Institute, National Institutes of Health, Rockville, MD 20850 USA; 6 Department of Internal Medicine, School of Medicine, University of New Mexico, Albuquerque, NM 87131 USA; 7 Cancer Control and Population Sciences, University of New Mexico Comprehensive Cancer Center, Albuquerque, NM 87131 USA

**Keywords:** Diesel exhaust, Endothelial cell dysfunction, Circulatory metabolites, Cardiovascular disease risk

## Abstract

**Background:**

Chronic exposure to diesel exhaust has a causal link to cardiovascular diseases in various environmental and occupational settings. Arterial endothelial cell function plays an important role in ensuring proper maintenance of cardiovascular homeostasis and the endothelial cell dysfunction by circulatory inflammation is a hallmark in cardiovascular diseases. Acute exposure to diesel exhaust in controlled exposure studies leads to artery endothelial cells dysfunction in previous study, however the effect of chronic exposure remains unknown.

**Results:**

We applied an ex vivo endothelial biosensor assay for serum samples from 133 diesel engine testers (DETs) and 126 non-DETs with the aim of identifying evidence of increased risk for cardiovascular diseases. Environmental monitoring suggested that DETs were exposed to high levels of diesel exhaust aerosol (282.3 μg/m^3^ PM_2.5_ and 135.2 μg/m^3^ elemental carbon). Surprisingly, chronic diesel exhaust exposure was associated with a pro-inflammatory phenotype in the ex vivo endothelial cell model, in a dose-dependent manner with CCL5 and VCAM as most affected genes. This dysfunction was not mediated by reduction in circulatory pro-inflammatory factors but significantly associated with a reduction in circulatory metabolites cGMP and an increase in primary DNA damage in leucocyte in a dose-dependent manner, which also explained a large magnitude of association between diesel exhaust exposure and ex vivo endothelial biosensor response. Exogenous cGMP addition experiment further confirmed the induction of ex vivo biosensor gene expressions in endothelial cells treated with physiologically relevant levels of metabolites cGMP.

**Conclusion:**

Serum-borne bioactivity caused the arterial endothelial cell dysfunction may attribute to the circulatory metabolites based on the ex vivo biosensor assay. The reduced cGMP and increased polycyclic aromatic hydrocarbons metabolites-induced cyto/geno-toxic play important role in the endothelial cell dysfunction of workers chronic exposure to diesel exhaust.

**Supplementary Information:**

The online version contains supplementary material available at 10.1186/s12989-022-00463-0.

## Introduction

Diesel exhaust has been classified as an established human carcinogen by International Agency for Research on Cancer and is a major source of traffic related airborne ultrafine particulate matter (PM_2.5_) in urban atmosphere [1–3]. People working at multiple occupational settings including underground mining, bridge and tunnel construction, trucking, railroad, etc., are regularly exposed to diesel exhaust [3]. Diesel exhaust consists of volatile gases and carbonaceous cores with the latter carrying organic and inorganic pollutants. Nanosize or ultrafine (< 100 nm) diesel exhaust could deposit deep and have long retention time inside the lung [3–6]. In addition to the lung injury, studies of chronic exposure to traffic related air pollution in general or diesel exhaust in particular has established a strong link with cardiovascular health implications in a variety of settings [7]. The diesel exhaust in miners study also established the dose–response between diesel exhaust exposure and ischemic heart disease mortality [7]. The underlying biological mechanisms may include systemic inflammatory response, a shift in autonomic balance, and possible translocation of particles into the circulatory system [8].

Arterial endothelial cell dysfunction influenced by circulatory inflammation is a hallmark in the initiation and progression of cardiovascular disease [9]. Endothelial cells are often activates by cytokines such as C-reactive protein (CRP) and TNF-α via the nuclear factor-kappa beta pathway, leads to de novo expression and presentation of key adhesion molecules and chemotactic factors in cell surfaces for the recruitment of leukocytes [10]. Compared to the endothelial cell activation, the suppression of endothelial cells may also represent the abnormal status, because the normal secretion and cell activition maintain the interaction with other circulatory cells including platelet to keep the integrity of endothelium [11]. A novel ex vivo endothelial biosensor assay has been developed to assess cell response with mRNA expression of key adhesion molecules and chemotactic factors in primary human coronary arterial endothelial cells (HCAECs) treated ex vivo with diluted serum samples from study subjects. This method has been validated by showing a greater potential for inducing endothelial cell adhesion molecules and chemokines in HCAECs treated with serum samples from patients with coronary artery disease or obstructive sleep apnea [12–14]. Endothelial cell activation was also identified to be elevated in subjects exposed to high levels of diesel exhaust (100 mg/m^3^) concomitant with nitrogen dioxide (500 ppb) in a controlled exposure study [15] or in Navajo community residents who live closer to abandoned uranium mine features versus who lives further away [16]. In addition, our previous study of carbon black packers identified endothelial cell dysfunction that was mostly driven by systemic inflammation with TNF-α having the largest effect [17]. All these studies provide strong evidence supporting ex vivo biosensor assay as a phenotypic biomarker associated with the early-stage of vascular diseases including atherosclerosis.

We have characterized a unique occupational cohort of diesel engine testers (DETs) who exam the performance of the newly assembled diesel-fueled vehicle engines for quality assurance in China [18–21]. Environmental monitoring at the workplace suggested that DETs were exposed to high levels of PM_2.5_ (282.3 μg/m^3^) and elemental carbon (135.2 μg/m^3^). In this study, we applied ex vivo endothelial biosensor assay to assess cumulative inflammatory potential in serum samples from 133 DETs and 126 non-DETs. In addition, the role of circulatory pro-inflammatory factors, metabolomics, and leucocyte DNA damage on the association between diesel exhaust exposure and endothelial biosensor response was assessed using mediation analyses. Finally, exogenous cGMP addition experiment was conducted to recapitulate the association between metabolite and biosensor response seen at the population level.

## Results

### Characteristics of study subjects

Diesel engine testing workshop has much higher levels of airborne PM_2.5_ and elemental carbon than reference area (Table 1). Accordingly, the levels of urinary metabolites (i.e., 2-OHFlu, 1-OHP, 1- & 2-OHNa, and 2- & 9-OHPh) in DETs were 2.4- to 4.4-fold higher than that seen in non-DETs (Table 1). The distribution of most demographic variables was comparable between the two groups except that DET smokers tended to smoke less than non-DET smokers. The average RNA yield from HCAEC cultures treated with sera from non-DETs and DETs was very similar (Table 1).

**Table 1:** Demographics and internal doses of diesel exhaust exposure in 133 DETs and 126 non-DETs

Variable	Non-DET	DET	*P* value
Age (y, mean ± SD)	32.0 ± 11.2	32.1 ± 8.7	0.89^a^
Sex (Male, %)	100	100	
Race (Han Chinese, %)	100	100	
Current smokers (n, %)	61, 48.4	79, 59.4	0.076^b^
Packyears (M, Q1–Q3)^d^	8 (4.2–20)	5 (2.5–10)	0.020^c^
BMI (mean ± SD)	23.7 ± 4.4	24.6 ± 3.4	0.087^a^
Occupational history (y, M, Q1–Q3)		8.5 (5.6–9.6)	
Total RNA (µg, M, Q1–Q3)	0.94 (0.62–2.51)	0.94 (0.47–1.94)	0.20
Urinary OH-PAHs (μg/g cr)			
2-OHFlu (M, Q1–Q3)	0.61 (0.29–0.94)	1.59 (1.02–2.40)	< 10^−4c^
1-OHP (M, Q1–Q3)	0.76 (0.23–1.36)	2.30 (1.28–3.44)	< 10^−4c^
1- & 2-OHNa (M, Q1–Q3)	2.07 (0.79–5.36)	5.06 (2.77–9.51)	< 10^−4c^
2- & 9-OHPh (M, Q1–Q3)	0.63 (0.34–1.31)	2.79 (1.82–4.16)	< 10^−4c^
PM2.5 (μg/m^3^, mean ± SD, n)	91.9 ± 3.4, 6	282.3 ± 111.3, 16	
PM2.5 associated EC (μg/m^3^, mean ± SD, n)	11.8 ± 0.6, 6	135.2 ± 56.6, 16	

Current smokers were defined as individuals who had smoked more than 100 cigarettes in their lifetime and continued to smoke during the period of interview or if quit, quit within 1 month prior to interview.

1-OHNa, one-hydroxynaphthalene; 2-OHNa, 2-hydroxynaphthalene; 2-OHFlu, 2-hydroxyfluorene; 2-OHPh, 2-hydroxyphenanthrene; 9-OHPh, 9-hydroxyphenanthrene; 1-OHP, 1-hydroxypyrene; M, median; Q, quartile; PM, particulate matter; EC, elemental carbon; SD, standard deviation

^a^Student’s t test

^b^Chi square test

^c^Rank sum test

^d^Values in current smokers

### Diesel exhaust exposure induced the ex vivo endothelial cell dysfunction

Principal component (PC) extracted from six urinary polycyclic aromatic hydrocarbons (PAH) metabolites reflective of diesel exhaust exposure was used as the internal dose to assess its association with ex vivo biosensor PC as a global index quantifying endothelial cells gene expression. A dose-dependent increase in ex vivo biosensor PC that did not vary by cigarette smoking status was identified in 124 DETs and 123 non-DETs with complete expression data for all seven biosensor genes, suggesting chronic exposure to a high level of diesel exhaust repressing endothelial cells associated gene expression (Table 2). Individual gene analysis identified CCL5 and VCAM that were more susceptible to be affected by diesel exhaust exposure with VCAM showing the largest reduction in mRNA expression (Table 2).

**Table 2:** Internal dose of disesel exhaust exposure and biosensor gene expressions in primary HCAECs treated with sera from 133 DETs and 126 non-DETs

Variable	Internal exposure category of disesel exhaust			Trend test^a^	
	Low	Medium	High	Estimate (95%CI)	*P* value
# non-DETs	77	41	9		
# DETs	10	47	79		
Urinary PC	~ − 0.67	− 0.67 to 0.18	0.18 ~		
Biosensor PC	− 0.24 (− 0.56 to 0.08)	0.02 (− 0.29 to 0.32)	0.29 (− 0.03 to 0.61)	0.27 (0.03 to 0.50)	0.025
Delta Ct					
CCL2	− 8.66 (− 9.17 to − 8.14)	− 8.35 (− 8.85 to − 7.86)	− 8.25 (− 8.76 to − 7.75)	0.20 (− 0.17 to 0.57)	0.289
CCL5	− 1.65 (− 1.93 to − 1.36)	− 1.50 (− 1.78 to − 1.23)	− 1.13 (− 1.41 to − 0.85)	0.26 (0.06 to 0.47)	0.013
CXCL8	− 8.99 (− 9.68 to − 8.31)	− 9.29 (− 9.95 to − 8.63)	− 9.13 (− 9.82 to − 8.44)	− 0.07 (− 0.57 to 0.43)	0.782
CXCL12	− 3.23 (− 3.56 to − 2.90)	− 3.01 (− 3.33 to − 2.70)	− 2.87 (− 3.19 to − 2.55)	0.18 (− 0.06 to 0.42)	0.135
ICAM	− 5.61 (− 5.98 to − 5.25)	− 5.30 (− 5.66 to − 4.95)	− 5.16 (− 5.52 to − 4.80)	0.23 (− 0.04 to 0.49)	0.095
SELP	0.10 (− 0.16 to 0.36)	0.19 (− 0.06 to 0.44)	0.47 (0.21 to 0.72)	0.19 (− 0.001 to 0.37)	0.052
VCAM	− 3.20 (− 3.66 to − 2.75)	− 2.75 (− 3.19 to − 2.31)	− 2.48 (− 2.93 to − 2.02)	0.36 (0.03 to 0.69)	0.032
Relative quantification					
CCL2	403.10 (282.28 to 575.23)	326.97 (231.84 to 461.12)	305.28 (214.67 to 434.14)	0.87 (0.67 to 1.13)	0.289
CCL5	3.13 (2.57 to 3.82)	2.83 (2.34 to 3.43)	2.18 (1.80 to 2.66)	0.83 (0.72 to 0.96)	0.013
CXCL8	509.52 (316.93 to 818.59)	626.86 (397.0 to 990.49)	561.83 (348.25 to 905.77)	1.05 (0.74 to 1.48)	0.782
CXCL12	9.37 (7.47 to 11.75)	8.08 (6.49 to 10.06)	7.31 (5.84 to 9.14)	0.88 (0.75 to 1.04)	0.135
ICAM	48.87 (37.92 to 62.94)	39.42 (30.87 to 50.39)	35.75 (27.78 to 46.01)	0.86 (0.71 to 1.03)	0.095
SELP	0.93 (0.78 to 1.12)	0.88 (0.74 to 1.04)	0.72 (0.61 to 0.86)	0.88 (0.77 to 1.00)	0.052
VCAM	9.22 (6.70 to 12.67)	6.72 (4.95 to 9.15)	5.57 (4.07 to 7.63)	0.78 (0.62 to 0.98)	0.032

Urinary PC was the first PC generated based on six urinary PAH metabolites and correlated strongly with diesel exhaust exposure (r > 0.64). Biosensor PC was extracted based on delta Ct of 7 ex vivo biosensor genes and explained 39% of total variance

^a^Generalized linear model was used to assess the association between internal dose of diesel exhaust exposure and ex vivo biosensor PC in 124 DETs and 123 non-DETs with adjustment for age, obesity, internal dose of cigarette smoking, and passage of cells. All significant associations did not vary by smoking status

### Serum-borne inflammatory factors in diesel exhaust chronic exposure population were not the mian cause of endothelial cell dysfunction

Our study in an occupational cohort of carbon black packers identified strong evidence of ex vivo activation of endothelial cells caused by inflammatory mediators in serum from carbon black packers [17]. Because of considerable similarity in primary sphere diameter, high surface area per mass values, and aciniform morphology between carbon black and diesel exhaust [22], we assessed whether the association between diesel exhaust exposure and endothelial cell dysfunction was mediate by circulatory inflammation factors. As shown in Additional file 1: Figure S1, there was no obvious change in cytokines (IL-1β and TNF-α) and chemokines MIP-1β in serum form chronic exposure to diesel exhaust, only decreased the levels of IL-6 (*P* = 0.0017) and IL-8 (*P* < 0.0001) in serum. Besides, three major PCs were extracted from the seven circulatory cytokines and chemokines with total variance explained over 70% (Additional file 1: Table S2). However, none of them were associated with ex vivo biosensor PC (Additional file 1: Table S3). Thus, the endothelial cell dysfunction due to diesel exhaust exposure was not mediated by circulatory pro-inflammatory factors.

### Circulatory metabolites in serum-borne after diesel exhaust chronic exposure mediated endothelial cell dysfunction

**Fig. 1:**
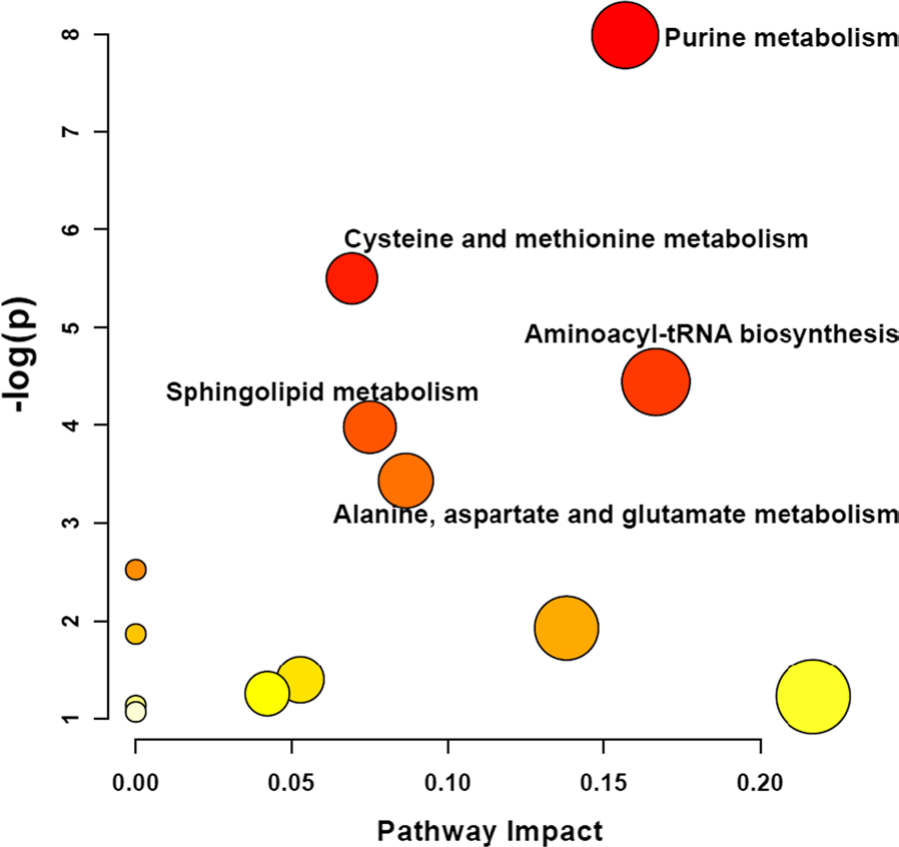
Pathway analysis based on targeted metabolomics study. Twenty two out of the 133 metabolites were associated with internal dose of diesel exhaust exposure with FDR < 0.05. Pathway analysis identified purine metabolism as a top pathway affected by diesel exhaust exposure (FDR = 0.029). Five metabolites (i.e., IMP, inosine, hypoxanthine, adenine, and cGMP) in the purine pathway had reduced measures associated with increasing diesel exhaust exposure. Additional four pathways including cysteine and methionine metabolism, Aminoacyl-tRNA biosynthesis, Sphingolipid metabolism, and Alanine, aspartate and glutamate metabolism seemed to be affected as well though with FDRs > 0.05

**Fig. 2:**
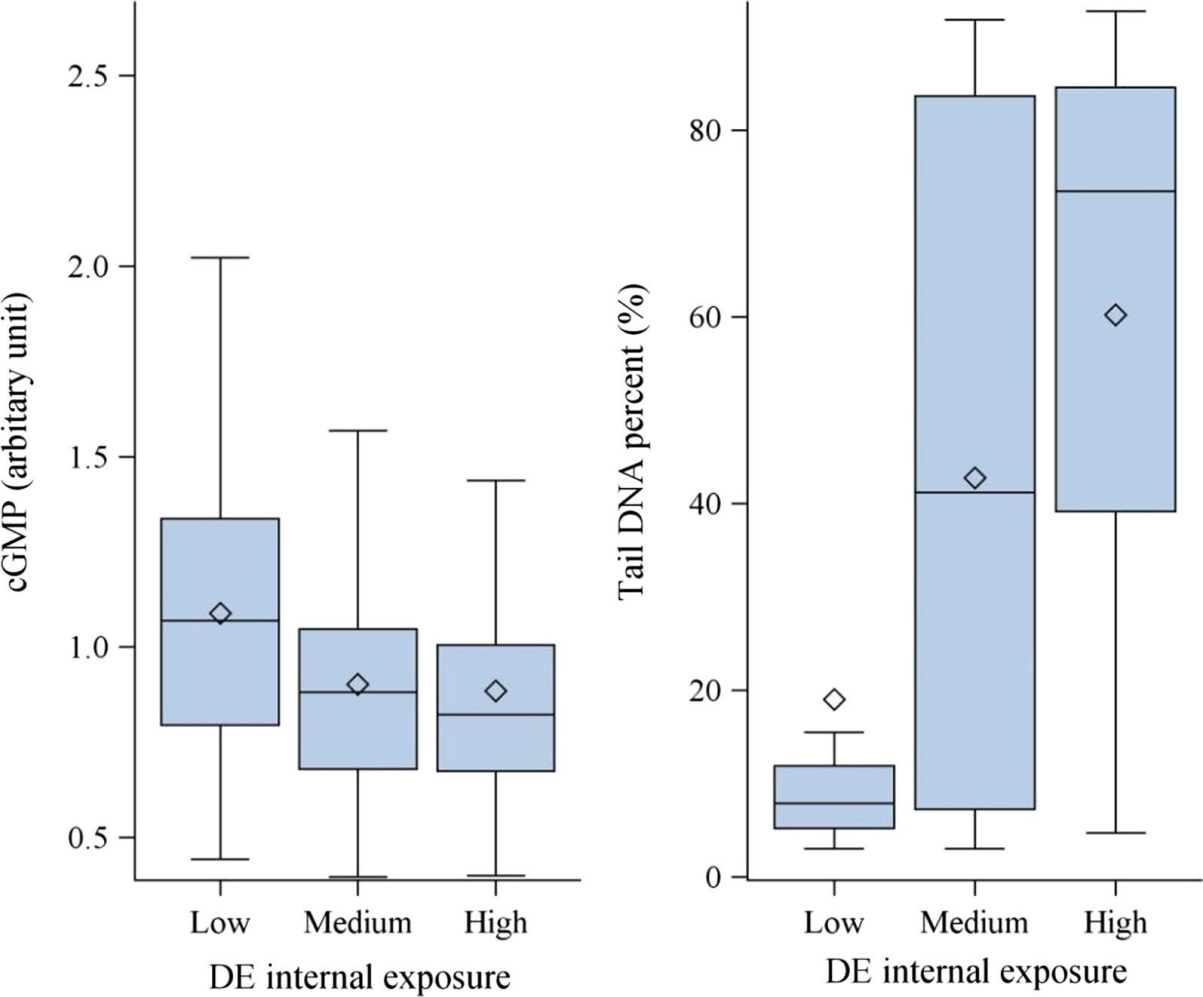
The distribution of circulatory cGMP and Tail DNA percent in study subjects with different internal diesel exhaust exposure. Study subjects were categorized into three groups including low, medium, and high according to their internal exposure to diesel exhaust. Dose–response were identified between exposure category and levels of cGMP and tail DNA percent. The five horizontal bars from bottom to top represent the minimum, first quartile, median, third quartile, and maximum. Symbol “◊” represents mean value

Targeted metabolomics detected 133 circulatory metabolites from study subjects. Twenty-two metabolites were identified to be associated with internal dose of diesel exhaust exposure with FDR < 0.05 (Table 3). Pathway analysis identified purine metabolism as a top pathway affected by diesel exhaust exposure (Fig. 1) which included IMP, inosine, hypoxanthine, adenine, and cGMP (Fig. 2), all showing reduction associated with increasing diesel exhaust exposure. Among these 22 metabolites, we only found cGMP associated with ex vivo biosensor PC with a nominal *P* value of 0.0081 when internal dose of diesel exhaust exposure was also included in the model (Additional file 1: Table S4). Further mediation analysis suggested that almost a quarter of magnitude of association between diesel exhaust exposure and ex vivo biosensor PC was explained by a reduction of cGMP due to diesel exhaust exposure (Table 4). Individual gene analysis suggested that all three genes, e.g., CCL5, SELP, and VCAM were affected by diesel exhaust exposure through reducing cGMP in circulation. In vitro functional validation analysis was conducted to assess whether exogenous cGMP could induce biosensor response when added to workers’ serum with lowest background level of cGMP and found mRNA expressions of CCL2, CCL5, and VCAM were significantly induced (Table 5).

**Table 3:** Internal dose of diesel exhaust exposure and plasma metabolites in 124 DETs and 123 non-DETs^a^

Metabolite	Estimate	SE	Fold of change	Raw *P*	FDR
S-adenosylhomocysteine	− 0.080	0.016	0.92	5.7 × 10^–7^	0.0001
3-Ketodihydrosphingosine	0.279	0.067	1.32	3.8 × 10^–5^	0.0025
Isoleucine	− 0.017	0.004	0.98	5.6 × 10^–5^	0.0025
IMP	− 0.244	0.062	0.78	0.0001	0.0037
Inosine	− 0.176	0.046	0.84	0.0002	0.0040
1-Methylhistidine	− 0.067	0.018	0.94	0.0002	0.0040
Carnitine C14	0.189	0.050	1.21	0.0002	0.0040
2-Aminooctanoic acid	0.136	0.038	1.15	0.0004	0.0062
Hypoxanthine	− 0.359	0.101	0.70	0.0005	0.0068
Indole-3-carboxylic acid	− 0.074	0.021	0.93	0.0005	0.0072
1-Methylnicotinamide	0.201	0.058	1.22	0.0006	0.0072
Carnitine C12	0.172	0.052	1.19	0.0010	0.0110
SDMA ADMA	− 0.051	0.016	0.95	0.0013	0.0132
Adenine	− 0.075	0.024	0.93	0.0016	0.0154
5'-Deoxy-5'-methylthioadenosine	− 0.089	0.029	0.91	0.0019	0.0169
N-Acetylaspartic acid	− 0.049	0.016	0.95	0.0021	0.0169
cGMP	− 0.092	0.030	0.91	0.0022	0.0172
Galactose	− 0.034	0.012	0.97	0.0035	0.0258
Asparagine	0.068	0.023	1.07	0.0040	0.0280
Carnitine C18	0.115	0.040	1.12	0.0043	0.0285
Acetylalanine	− 0.031	0.011	0.97	0.0058	0.0361
Serine	0.061	0.022	1.06	0.0067	0.0386

^a^Internal dose of diesel exhaust exposure was defined as the urinary PC extracted from four urinary metabolites which has shown an excellent association with diesel exhaust exposure status. This PC was converted into an ordered categorical variable with three values (0, 1, 2) with each group having same number of subjects to calculate the estimate for the dose–response. Association between internal dose of diesel exhaust exposure and natural-log transformed serum metabolite levels was assessed using generalized linear model with adjustment for age, obesity, and internal dose for cigarette smoking. Fold of change was the exponential of the estimate

**Table 4:** Mediation effect of serum cGMP level on the association between diesel exhaust exposure and biosensor responses in 124 DETs and 123 non-DETs

Mediator	Biosensor	Mediator biosensor asso^a^			Diesel biosensor asso^a^			Mediation effect^b^	
		Est (b)	SE	Raw *P*	Est (c')	SE	Raw *P*	PM	*P* perm
Individual mediator									
cGMP	Biosensor PC1	− 0.669	0.251	0.008	0.204	0.119	0.087	0.232	0.02
	CCL5	− 0.555	0.228	0.016	0.210	0.106	0.049	0.196	0.01
	ICAM	− 0.616	0.294	0.037	0.167	0.136	0.222	0.253	0.055
	SELP	− 0.460	0.207	0.027	0.144	0.096	0.136	0.228	0.01
	VCAM	− 0.858	0.367	0.020	0.283	0.170	0.097	0.218	0.025
Tail DNA %^c^	Biosensor PC1	0.062	0.030	0.042	0.110	0.138	0.423	0.528	0.035
	CCL5	0.074	0.027	0.007	0.119	0.122	0.332	0.553	0.005
	ICAM	0.058	0.035	0.102	0.038	0.159	0.811	0.752	0.055
	SELP	0.033	0.025	0.182	0.088	0.112	0.432	0.427	0.07
	VCAM	0.109	0.045	0.016	0.093	0.202	0.645	0.699	0.02
Double mediator^c^									
cGMP	Biosensor PC1	− 0.607	0.274	0.028	0.078	0.137	0.572	0.667	0.015
Tail DNA %	Biosensor PC1	0.051	0.030	0.095					
cGMP	CCL5	− 0.589	0.248	0.018	0.085	0.122	0.485	0.679	< 0.005
Tail DNA %	CCL5	0.065	0.027	0.019					
cGMP	ICAM	− 0.565	0.324	0.082	0.005	0.160	0.977	0.970	0.025
Tail DNA %	ICAM	0.049	0.036	0.172					
cGMP	SELP	− 0.487	0.227	0.033	0.061	0.112	0.587	0.607	0.015
Tail DNA %	SELP	0.025	0.025	0.311					
cGMP	VCAM	− 0.799	0.410	0.052	0.047	0.202	0.814	0.846	0.005
Tail DNA %	VCAM	0.096	0.045	0.035					

^a^Internal dose of diesel exhaust exposure was defined as urinary PC extracted from six urinary metabolites which has shown an excellent association with diesel exhaust exposure status. This PC was converted into an ordered categorical variable with three values (0, 1, 2) with each group having same number of subjects to calculate the estimate for the dose–response. Generalized linear model was used to assess association (c') between internal dose of diesel exhaust exposure and ex vivo biosensor PC with age, obesity, internal of for cigarette smoke exposure, passage of cells, and mediators (e.g., natural-log transformed serum metabolite levels or tail DNA %, b) as covariate for adjustment

^b^The proportion mediated effect size that quantifies the proportion of a total effect mediated was calculated using the following equation: ab / (ab + c'). The database was permuted for 200 times to generate a null distribution of a*b. *P*_perm_ was calculated as the number of permuted databases generating an a*b that is greater than observed value divided by 200. For analyses involving double mediators, sum of a*b was calculated

^c^Any analyses involving Tail DNA% were conducted in 105 DETs and 119 non-DETs. Metabolite biosensor association was quantified with every 10% increase in tail DNA %

**Table 5:** The effect of cGMP on expression of individual biosensor genes from the exogenous cGMP addition experiment

Biosensor gene	Variable	Unit of change	Estimate (95%CI)^a^	RQ^b^	*P*^a^
CCL2	cGMP (pmol/ml)	50	− 0.32 (− 0.62 to − 0.01)	1.25	0.043
CCL5	cGMP (pmol/ml)	50	− 1.53 (− 2.43 to − 0.63)	2.88	0.0025
VCAM	cGMP (pmol/ml)	50	− 0.45 (− 0.79 to − 0.12)	1.37	0.011

^a^Generalized linear model was used to quantify the change of expression (Delta Ct) of biosensor genes by addition of cGMP in the medium in exogenous cGMP addition experiment with adjustment for serum ID. The slopes of linear curves between gene expression (delta Ct) and cGMP levels were of no difference between serum samples from three individuals (all *P*s > 0.07)

^b^Relative quantification was calculated as RQ = 2^−(estimate)^

### Enhanced DNA damage in circulatory blood lymphocytes leaded to the endothelial cell dysfunction

**Fig. 3:**
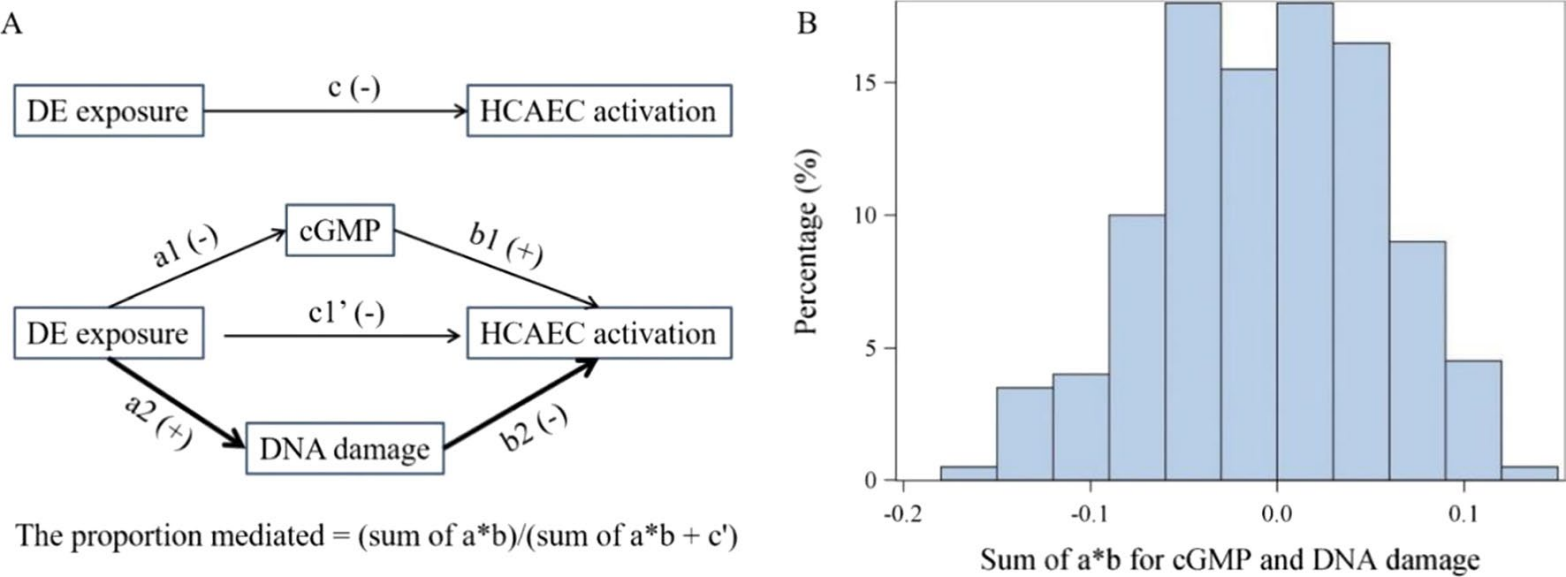
Circulatory cGMP and DNA damage mediating the effect of diesel exhaust exposure on HCAEC activation. In mediation analysis (**A**), the c coefficient denotes the direct effect of diesel exhaust exposure on HCAEC activation, without controlling for circulatory cGMP and DNA damage (mediators). HCAEC activation was expressed as RQ. The c' coefficient denotes the direct effect of diesel exhaust exposure on HCAEC activation, controlling for two mediators. The proportion mediated is equal to (sum of a*b)/(sum of a*b + c'). We took a permutation-based method to assess whether the proportion mediated was statistically significant or not (**B**). The relationship between CCL5 expression and the vector of independent variables was permuted for 200 times. Each permutated database allowed the association analysis of cGMP or DNA damage with internal dose of diesel exhaust exposure and other covariates to calculate a and of CCL5 expression with internal dose of diesel exhaust exposure and other covariates without and with including mediators to calculate the c, b, and c'. Permutation was conducted for 200 times to generate the distribution of sum of a*b under null hypothesis of no mediation. Value of sum of a*b calculated using observed data was compared to the distribution generated by permutation and *P*_perm_ was calculated as the number of permuted databases generating sum of a*b that is greater than observed value (0.18) divided by 200

Diesel exhaust contains multiple cyto/geno-toxicants whose reactive metabolites and byproducts (e.g., reactive oxygen species [ROS]) could cause various types of DNA damages. Our previous cohort studies of DETs had detected dose-dependent increases of primary DNA damage (Fig. 2) and cytogenetic changes in peripheral blood and etheno-DNA adduct excreted in urine [23–25]. Unrepaired DNA damage lesions accumulated due to exposure to exogenous insults or endogenous metabolites could lead to a growing set of pathophysiological changes that may interactively contribute to progressive vascular aging [26, 27]. Thus, we hypothesized that DNA damage may mediate chronic diesel exhaust exposure induced endothelial cell dysfunction ex vivo. Primary DNA damage level (e.g., tail DNA %) detected in peripheral blood using comet assay was selected to assess the DNA damaging capacity of the serum because this endpoint is more towards formation of DNA damage versus genomic instability outcomes after repair. Interestingly, tail DNA % explained over half of the magnitude of association between diesel exhaust exposure and ex vivo biosensor PC with CCL5 and VCAM as the most affected genes (Table 4). We further conducted mediation analyses including two mediators (e.g., cGMP and primary DNA damage) and found a significant combinational effect of these two factors in alleviating the association between diesel exhaust exposure and endothelial cell dysfunction with PMs ranging from 0.61 to 0.97 (all *P*s < 0.03, Table 4 and Fig. 3). To further confirm the possible mechanisms of ex vivo biosensor cell dysfunction, we randomly selected 15 circulatory s derived from the chronic exposure to diesel exhaust in subjects and conducted the ex vivo biosensor assay with followed 2*150 bp paired-end sequencing (PE150) on an Illumina NovaseqTM 6000 platform. The differentially expressed genes (DEGs) between the diesel exhaust exposure and control groups exhibited the apoptosis-related genes were up-regulated after diesel exhaust exposure (Additional file 1: Table S5), which further verified the endothelial cells dysfunction in the ex vivo biosensor assay may attribute to the metabolites of diesel exhaust exposure induced cytotoxicity.

## Discussion

We applied a novel ex vivo endothelial biosensor assay to assess the capacity of serum samples from workers with chronic exposure to high levels (282.3 mg/m^3^) of diesel exhaust in induction of mRNA expression of key adhesion molecules and chemotactic factors in coronary arterial endothelial cells, a hallmark event in cardiovascular homeostasis by regulating blood fluidity and fibrinolysis, vascular tone, monocyte/leukocyte adhesion, and platelet aggregation [28, 29]. Due to the ethical issues involved in population studies, the difficulty of obtaining vascular tissue from research subjects, and the inability to monitor vascular endothelial cells or vascular function in vivo, we used the ex vivo biosensor assay to evaluate the effect of serum taken from the body on primary vascular endothelial cells, which is more beneficial for us to explore the mechanism of systemic effects (especially vascular effects) induced by pulmonary exposed to diesel exhaust. Surprisingly, instead of increasing expression of ICAM and VCAM as seen in controlled exposure of healthy volunteers to high dose diesel exhaust (106 mg/m^3^) or diesel exhaust constituent NO_2_ (500 ppb) for 2 h [15], chronic diesel exhaust exposure inhibited expressions of several ex vivo endothelial biosensor genes in a dose-dependent manner with CCL5 and VCAM being affected the most. Because this ex vivo endothelial biosensor assay has been validated as a holistic biomarker reflective of total inflammatory potential of serum from patients with coronary artery disease or obstructive sleep apnea [12, 13], our results suggested that DETs may have reduced inflammation levels in their serum. Our findings were consistent with two geographically independent cross-sectional studies that chronic occupational exposure to high dose pure primary diesel exhaust was associated with reduced levels of circulatory pro-inflammatory cytokines and chemokines [18, 30, 31].

The assay used in this study was fully optimized and we took a very comprehensive quality assurance strategy as detailed in the method section for conducting the entire experiment. The reliability of this assay was also indicated by the low coefficients of variation (e.g., 2.8% to 11.1%) for Cts of eight genes in quality control samples being measured together with each batch of assays across the entire experiments. This assay has been shown to be sufficiently sensitive to detect the inflammatory potential of serum from healthy volunteers with inhalation exposure to diesel exhaust or NO_2_ for only 2 h [15] or from Navajo community residents whose residential proximity to abandoned uranium mine features was in linear dose–response to biosensor gene expressions [16]. For example, Campen's group have repoted that short-term exposure to high dose diesel exhaust significantly increased the levels of inflammatory cytokines (especially IL-8) in serum and promoted the injury of isolated vascular endothelial cells, based on the ex vivo biosensor assay [15]. Moreover, serum samples from carbon black packers who were exposed to a high level of nano-scale carbon black aerosol (800.0 μg/m^3^) and non-exposed controls were tested using the ex vivo biosensor assay during the period when this occupational exposure study was conducted [17]. Serum from carbon black packers induced a 1.31- to 9.06- fold of increase in expressions of ICAM, CCL2, CCL5, CXCL8, and VCAM compared to serum from non-exposed controls which were mostly explained by circulatory pro-inflammatory factors such as TNF-α, IL-1β, and IL-6 [17]. Because samples from both studies were randomized and assayed together using exact same procedure and reagents, we are confident that the results seen in DETs versus non-DETs were valid and free of technical flaws. Consistent with findings from others [12], serum HDL level was significantly associated with reduction of biosensor gene expressions with VCAM (*P* = 0.05), CXCL12 (*P* = 0.03), and CCL5 (*P* = 0.02) as the most affected genes in our study. The ex vivo endothelial biosensor model provides confidence in the role of HDL dysfunction in endothelial cell dysfunction. Carbon black was often studied as a carbonaceous core analog of diesel exhaust due to considerable similarity in particle morphology [19, 31, 32]. Carbon black as a typical manufactured nanoscale particle consisting of pure elemental carbon is an occupational respiratory hazard commonly seen in runner industry [33]. Thus, the striking difference in inducing endothelial cell dysfunction ex vivo between carbon black and diesel exhaust exposures may be largely due to the effects of organic components and gases in diesel exhaust.

Exogenous and endogenous metabolites with its downstream oxidative stress have been recognized as the common underlying cellular mechanism for the development of endothelial dysfunction among many risk factors for atherosclerosis [34, 35]. In this study, primary DNA damage detected in peripheral blood using alkaline comet assay could result from the oxidative stress and reactive cyto/geno-toxic metabolites in circulation. However, instead of increasing endothelial cell activation, DNA damage was associated with reduced expression of biosensor genes and its inclusion in the mediation model almost completely negated the effect of diesel exhaust exposure on ex vivo biosensor response. Our previous study of DETs had identified increased levels of genomic instability and cell death and inhibition of cell division in blood cultures stimulated with phytohaemagglutinin in vitro [21, 36]. Thus, a most probable explanation would be that oxidative stress and geno-toxicants in circulation due to exposure to high levels of diesel exhaust may be robust enough to cause dramatic DNA damage and cytotoxicity that subsequently inhibit multiple biological processes. This premise may also explain the strikingly different observations made between our study and controlled human exposure experiment [15] that two-hour exposure to high dose diesel exhaust would not able to elevate oxidative stress and cyto/geno-toxicants such as reactive metabolites of polycyclic aromatic hydrocarbons and nitroarenes [37] in blood to a level sufficient enough for causing DNA damage and cytotoxicity both in vivo and in vitro*.* Although under chronic exposure to high diesel exhaust levels endothelial cell injuries caused by geno-toxic and cyto-toxic metabolites lead to endothelial cell detachment and apoptosis contributing to the endothelial dysfunction and progression to cardiovascular disease, the detailed mechanism is still need future exploration in cohort or animal models.

Our targeted metabolomics provides optimal measurement of 133 chemically characterized and biochemically annotated metabolites in our plasma samples, among which 22 metabolites had significant (FDR < 0.05) association with diesel exhaust exposure. Circulatory cGMP level inversely associated with diesel exhaust exposure (FDR = 0.017) was the only metabolite associated with increased expression of biosensor genes. More importantly, the reduction of biosensor gene expressions associated with increasing diesel exhaust exposure was partially mediated by circulatory cGMP level. Our exogenous cGMP addition experiment further confirmed addition of cGMP in culture medium at physiological levels significantly increased the expressions of CCL2, CCL5, and VCAM. However, our research was different from the results of Kovalski et al., Lo et al. and Muraki et al. studies [38–40], which may be caused by different research methods. Their studies focused on the regulation of endogenous cGMP in vivo, while our study was the in vitro experiment of adding exogenous cGMP. The regulation of inflammatory response by cGMP in vivo is a very complex process. Besides, the cGMP synthesized by guanylate cyclase enzyme in endothelial cells was a key second messenger molecule in endothelium biology that commuted the vasodilation signal of nitric oxide (NO) produced in endothelial cells to the vascular smooth muscle cells [41]. Thus, lower cGMP levels in circulation due to diesel exhaust exposure may lead to vasodilator dysfunction that causes smooth muscle cell dysfunction, increased vascular stiffness, and accelerated vascular cell senescence, all contributing to the development of cardiovascular disease [42]. In addition to its vasodilation function in vascular smooth muscle cells, our findings also suggested that cGMP may actually affect the endothelial cells in an autocrine manner and induce gene expressions in HCAECs. Interestingly, we also found that circulatory cGMP level was inversely correlated with DNA damage in blood cells (spearman correlation = -0.16, P = 0.018 with adjustment for age, obesity, cigarette smoking, and diesel exhaust exposure), suggesting that cGMP’s association with biosensor response may be partially explained by its correlation with DNA damage as well. Indeed, cGMP deficiency may impair DNA repair and contribute to genomic instability in healthy tissue potentially through metabolic reprogramming from oxidative phosphorylation to glycolysis which increases ROS production and oxidative DNA damage [43].

This study was hypothetically designed to use endothelial biosensor assay to identify ex vivo evidence of endothelial cell biomarkers changes that may contribute to the increased cardiovascular incidence and mortality in diesel exhaust exposed populations. However, mechanisms independent of circulatory inflammation mediated endothelial cell biomarker change were suggested and involved cGMP signaling and DNA damage response that may contribute to cardiovascular toxicity and health effects induced by chronic exposure to high levels of diesel exhaust. Similarly, Campen's group [44] found that multi-walled carbon nanotube (MWCNT) exposure trigged significant upregulation of lung metalloproteinases MMPs, ADAMs, and ADAMTSs and released protease-generated peptides (such as TSP_402-46_) could enter the circulatory system to induce cell-surface receptor mediated systemic inflammation (the endothelial cell inflammatory marker of *Ccl2, Vcam1, Icam1* and *Tnfα* were significantly increased). Further confirmation of TSP_402–460_ peptide (as non-cytokines)-mediated model of systemic effects and vascular dysfunction following pulmonary nanoparticle exposure by synthesizing the TSP_402–460_ peptide and applying it in a CD36/CD47-mediated endothelial cell in vitro wound healing angiogenesis assay. The vascular endothelium is a complex organ that interacts with and responds to multiple environmental stimuli [45]. In addition to etiological contribution of endothelial cell abnormal status to pathogenesis of early-stage cardiovascular disease, similar mechanism was also involved in defense response triggered by pathogenic infection or injury [45]. Thus, the suppression of endothelial cell tested ex vivo by sera from diesel exhaust exposed workers may suggest a compromise in mounting an efficient immunological response towards systemic or localized infection. Indeed, a study of 95 million Medicare inpatient claims in the United States during 2000 to 2012 identified positive associations between short term exposure to PM_2.5_ and risk of hospital admission for 33 disease groups among which septicemia and skin and subcutaneous tissue infections were identified [46].

## Conclusion

In this study, an ex vivo biosensor assay was performed to evaluate the endothelium effect after chronic diesel exhaust exposure. The endothelial cell dysfunction may attribute the circulatory metabolites (cGMP and PAH metabolites) rather than circulatory inflammation. In the future study, more efforts should be devoted to evaluated the endothelial cell death mechanism involved in the cardiovascular disease after the chronic diesel exhaust exposure.

## Methods

### Study cohort and sample collection

The diesel engine tester (DET) study was established in 2012 by DETs from a diesel engine manufacturing plant and non-DETs controls from a water utility authority in the same city. Detailed description of the DET cohort and the working environment was introduced before [18–21]. In total, the diesel exhaust exposed group included 137 male DETs who had been testing heavy-duty diesel engines for at least six months prior to the enrollment into this study. The non-DET group consisted of 127 male workers from a local water authority (Table 1). The exclusion criteria included being chronic diseases (such as acute infection and cancer), or being exposed to X-ray in the past three months. Written informed consent was acquired from all participants prior to the interview and any procedures. The research protocol was approved by the Research Ethics Committee of the National Institute for Occupational Health and Poison Control, Chinese Center for Disease Control and Prevention.

We obtained demographic information, including age, sex, height, weight, race, smoking habit and occupational history of exposure at eligible participants. Each participant was asked to provide 4 mL venous blood and 50 mL urine for follow-up experiments.

### Diesel exhaust exposure

Diesel exhaust exposure was assessed using environmental monitoring of PM_2.5_ and its associated elemental carbon and PAH metabolites in end-shift urine after four consecutive working days. Detailed methodology and quality assurance have been described in previous study [18–21, 36]. Briefly, the pre-weighed Teflon filters (90 mm diameter, Millipore, USA) was used for airborne samples collection on 2 consecutive work days (8 h per work day) within one week before blood sample collection. The PM_2.5_ was analyzed using the gravimetric method. The EC was determined by using a Carbon Analyzer (DRI2001A, Atmoslytic Inc., USA) according to National Instisute for Occupational Safety and Health method 5040 (NIOSH5040). The high-performance liquid chromatography (HPLC) with fluorescence detector was used to measure the PHA constituents in collected PMs, based on the guidelines of OSHA No. 58.

### Cell culture and ex vivo biosensor assay

Primary HCAECs (ATCC PCS-100–020, Manassas, USA) between passages 4–6 were used to complete the biosensor assay in this study. Briefly, confluent HCAEC cultures were serum-starved with basal media for 24 h prior to incubation with basal medium containing 10% serum obtained from study subjects for 4 h at 37 °C. Each plate of cells was treated with proportional number of non-DETs and DETs in a randomized and blind fashion. Seven target genes including CCL2 (Hs00234140_m1), CCL5 (Hs00982282_m1), CXCL8 (Hs00174103_m1), CXCL12 (Hs00171022_m1), ICAM (Hs00164932_m1), SELP (Hs00174583_m1), and VCAM (Hs01003372_m1) with TBP (Hs00427620_m1) as the endogenous control gene were selected based on the involvement of expressed proteins in chemotaxis and adhesion of leukocytes on the surface of endothelial cells upon activation [9]. Two serum pools were established by combining serum aliquots from 15 non-DETs and 10 DETs, respectively, and were assayed with each batch of experiments as quality assessment samples. Coefficients of variation for cycle thresholds (Ct) of eight genes ranged from 2.8% to 11.1% with an average of 6.7%.

### Metabolite extraction and targeted metabolomics

Sixty microliters of human plasma were added to 240 μL of methanol (pre-cooled in − 80 °C) followed by vigorous vortexing and centrifugation at 4 °C for 15 min. The supernatant was divided to 100 μL per tube and evaporated to dryness using a SpeedVac concentrator (Thermo Savant). QC (quality control) samples were prepared by pipetting 10 μL of individual plasma samples and mixed together followed by the same extraction steps as above. Dried metabolites were reconstituted in 250 μL of 0.03% formic acid, vortexed, centrifuged at 15,000*g* for 15 min at 4 °C and the supernatant was analyzed using liquid chromatography–tandem mass spectrometry (LC–MS/MS). An Ultra Performance Liquid Chromatograph (UPLC) system (Waters, ACQUITY UPLC I-Class) was used for liquid chromatography, with an ACQUITY UPLC HSS-T3 UPLC column (150 × 2.1 mm, 1.8 μm, Waters) and the following gradient: 0–3 min 99% mobile phase A; 3–15 min 99–1% A; 15–17 min 1% A; 17–17.1 min 1–99% A; 17.1–20 min 99% A. Mobile Phase A was 0.03% formic acid in water. Mobile Phase B was 0.03% formic acid in acetonitrile. The flow rate was 0.25 ml·min-1, the column was at 35 °C and the samples in the autosampler were at 4 °C. The injection volume was 10 μL. Mass spectrometry was performed with a triple quadrupole mass spectrometer (Waters Xevo TQ-XS) in multiple reaction monitoring (MRM) mode. A total of 234 metabolites were monitored with 152 ion transitions in positive mode and 82 ion transitions in negative mode. Chromatogram review and peak area integration were performed using Skyline 4.2.0. 19037 (University of Washington). QC samples were injected every 10 samples throughout the analysis to monitor the instrument stability and normalize inter-batch variation. The peak area for each detected metabolite was normalized against the total ion count (TIC) of that sample. The TIC-normalized data of each metabolite was further normalized by that in QC samples to correct inter-batch variation. Normalized peak areas were used as variables for multivariate and univariate statistical data analyses. Hierarchical clustering was performed using Morpheus (https://software.broadinstitute.org/morpheus). PCA (principle component analysis), PLS-DA (partial least squares discriminant analysis) and variable importance in projection analysis were done using SIMCA-P 14.1 (Umetrics). Pathway analysis was performed using Metaboanalyst 4.0 (Chong et al. 2018). Generalized linear model (GLM) was used to assess the association between internal dose of diesel exhaust exposure and natural log transformed circulatory metabolites with adjustment for age, obesity, and internal dose for cigarette smoke exposure. False discovery rate was calculated to address the multiple comparison issue.

### Circulatory pro-inflammatory markers

Three pro-inflammatory cytokines (i.e., IL-1β, IL-6, and TNF-α) and two chemokines (i.e., IL-8 and MIP-1β) were measured in serum using cytometric bead array (BD Biosciences, USA). Serum C-reactive protein (CRP) was measured with an immunoturbidimetric assay (DiaSys, Germany). Detailed process and quality assurance are followed by the instruction manual as described previously [31].

### Alkaline comet assay

DNA damage in whole blood leucocytes was determined by the alkaline Comet assay system developed by Trevigen Inc with a slight modification [23]. Briefly, 30 μL pre-treated blood suspensions was added into 20-well Comet Slide and gelled at 4℃. Then, the slides were lysis with cold lysis solution and immersed in buffer F twice. Subsequently, the slides were transferred to an electrophoresis system. To quantify DNA damage, percentage of DNA in comet tail was quantified from 100 cells per subject with median as the value representing the DNA damage level in each subject.

### In vitro functional validation

Verified primary human umbilical vein endothelial cells (hUVEC) were seeded in 24-well plates and cultured to a 70–80% confluence. The cells were serum-starved for 2 h and then incubated with medium supplemented with 10% serum obtained from workers with low-level cyclic guanosine monophosphate (cGMP) spiked with different concentrations (0, 1, 10, 100 pmol/ml) of cGMP (Sigma G7504) for interference. The highest concentration of cGMP is about twofold higher than the maximum value of the normal range in serum seen in general populations. After 4 h incubation, total RNA was extracted and reverse transcribed to complementary DNA. qPCR was performed using the TaqMan assays described above.

### Statistical analysis

First, GLM was used to assess the association between diesel exhaust exposure and HCAECs activation with adjustment of age, obesity, cigarette smoking, and passage of cells. Our previous study has identified two principal components (PC) based on six urinary PAH metabolites with the first and second PCs strongly correlated with diesel exhaust exposure (r > 0.64) and cigarettes per day (r > 0.66), respectively [20]). Thus, these two PCs were used as an index of internal exposure for diesel exhaust and cigarette smoke, respectively. Principal component analysis (PCA) was conducted to extract a major PC (e.g., ex vivo biosensor PC) based on delta Ct of 7 biosensor genes that explained 39% of total variance (Additional file 1: Table S1). Thus, ex vivo biosensor PC was defined as a global index reflective of the magnitude of HCAECs activation and was assessed in association with diesel exhaust exposure first to minimize the issue of multiple comparisons. Individual gene association with diesel exhaust exposure was further assessed with delta Ct as the outcome to explore which biosensor gene was more responsive to diesel exhaust exposure. Second, the difference of magnitude of the association between models with (c′) and without (c) adjustment for potential mediators (Fig. 3A) was calculated to quantify the mediation effect of mediators on the association between diesel exhaust exposure and HCAECs activation [47]. A permutation-based method was used to assess whether the proportion mediated was statistically significant or not and was detailed in figure legend of Fig. 3B. Circulatory pro-inflammatory factors, metabolomics, and leucocyte DNA damage were assessed for their mediation effects. Third, GLM was used to quantify the change of expression (delta Ct) of biosensor genes by cGMP treatment in the medium in an exogenous cGMP addition experiment with adjustment for serum ID. An interaction term between treatments and serum ID was included in the GLM to assess whether the slopes of linear curves between gene expression and levels of added cGMP were of any difference between serum samples from three individuals. Plots were made using SAS ODS Graphics Designer. All statistical analyses were conducted using SAS (version 9.4, NC, USA, site 70080753).

## Supplementary Information

**Additional file 1: Figure S1.** Comparison of serum pro-inflammatory cytokines and chemokines between 125 DETs and 126 non-DETs. (A) IL-1β (*P* = 0.4684). (B) IL-6 (*P* = 0.0017). (C) IL-8 (*P* < 0.0001). (D) MIP-1β (*P* = 0.1176). (E) TNF-α (*P* = 0.0594). (F) MCP1 (*P* = 0.2468). Wilcoxon signed-rank test was used to compare values from both groups. **Table S1.** Principal component analysis of 7 biosensor genes in 124 DETs and 123 non-DETs (delta Ct). **Table S2.** Principal component analysis of blood cytokine and chemokine in 125 DETs and 126 non-DETs. **Table S3.** Pattern of triangles between diesel exhaust exposure, serum inflammation, and biosensor responses in 117 DETs and 122 non-DETs. **Table S4.** Pattern of triangles between diesel exhaust exposure, plasma metabolites and biosensor responses in 124 DETs and 123 non-DETs. **Table S5.** Expression of apoptosis-related genes in ex vivo biosensor cells treated with the plasma of diesel exhaust exposure workers.

## Data Availability

Not applicable.
