# Supplementary material for: Circulatory metabolites trigger ex vivo arterial endothelial cell dysfunction in population chronically exposed to diesel exhaust
Source: Part Fibre Toxicol. 2022 Mar 22;19:20. doi: 10.1186/s12989-022-00463-0 (PMC8939222; doi:10.1186/s12989-022-00463-0)
Supplement: Supplementary file 1 — Additional file 1: Figure S1. Comparison of serum pro-inflammatory cytokines and chemokines between 125 DETs and 126 non-DETs. (A) IL-1β (P = 0.4684). (B) IL-6 (P = 0.0017). (C) IL-8 (P < 0.0001). (D) MIP-1β (P = 0.1176). (E) TNF-α (P = 0.0594). (F) MCP1 (P = 0.2468). Wilcoxon signed-rank test was used to compare values from both groups. Table S1. Principal component analysis of 7 biosensor genes in 124 DETs and 123 non-DETs (delta Ct). Table S2. Principal component analysis of blood cytokine and chemokine in 125 DETs and 126 non-DETs. Table S3. Pattern of triangles between diesel exhaust exposure, serum inflammation, and biosensor responses in 117 DETs and 122 non-DETs. Table S4. Pattern of triangles between diesel exhaust exposure, plasma metabolites and biosensor responses in 124 DETs and 123 non-DETs. Table S5. Expression of apoptosis-related genes in ex vivo biosensor cells treated with the plasma of diesel exhaust exposure workers. [file 12989_2022_463_MOESM1_ESM.docx]

**Supplemental materials**

**Circulatory metabolites trigger *ex vivo* arterial endothelial cell dysfunction in population chronically exposed to diesel exhaust**

Wenting Cheng^1*^, Huanhuan Pang^2*^, Matthew J. Campen^3^, Jianzhong Zhang^1^, Yanting Li^1^, Jinling Gao^1^, Dunqiang Ren^4^, Xiaoya Ji^1^, Nathaniel Rothman^5^, Qing Lan^5^, Yuxin Zheng^1^, Shuguang Leng^6,7#^, Zeping Hu^2#^, Jinglong Tang^1#^

^1^ Department of Occupational and Environmental Health, School of Public Health, Qingdao University, Qingdao, Shandong, China 266021

^2^ School of Pharmaceutical Sciences, Tsinghua University, Beijing, China 100084

^3^ Department of Pharmaceutical Sciences, College of Pharmacy, University of New Mexico, Albuquerque, New Mexico, USA 87131

^4^ Department of Respiratory Medicine, Affiliated Hospital of Medical College of Qingdao University, Qingdao University, Qingdao, Shandong, China 266021

^5^ Division of Cancer Epidemiology and Genetics, National Cancer Institute, National Institutes of Health, Rockville, Maryland, USA 20850

^6^ Department of Internal Medicine, School of Medicine, University of New Mexico, Albuquerque, New Mexico, USA 87131

^7^ Cancer Control and Population Sciences, University of New Mexico Comprehensive Cancer Center, Albuquerque, New Mexico, USA 87131

* Co-first author

# Co-correspondence author

**Correspondence to** Shuguang Leng, Department of Internal Medicine, School of Medicine, University of New Mexico, Albuquerque, USA 87131. E-mail: [sleng@salud.unm.edu](mailto:sleng@salud.unm.edu); or Zeping Hu, School of Pharmaceutical Sciences, Tsinghua University, Beijing, China 100084. E-mail: [zeping_hu@tsinghua.edu.cn](mailto:zeping_hu@tsinghua.edu.cn); or Jinglong Tang, School of Public Health, Qingdao University, Qingdao, China 266021. Email: tangjinglong@qdu.edu.cn.


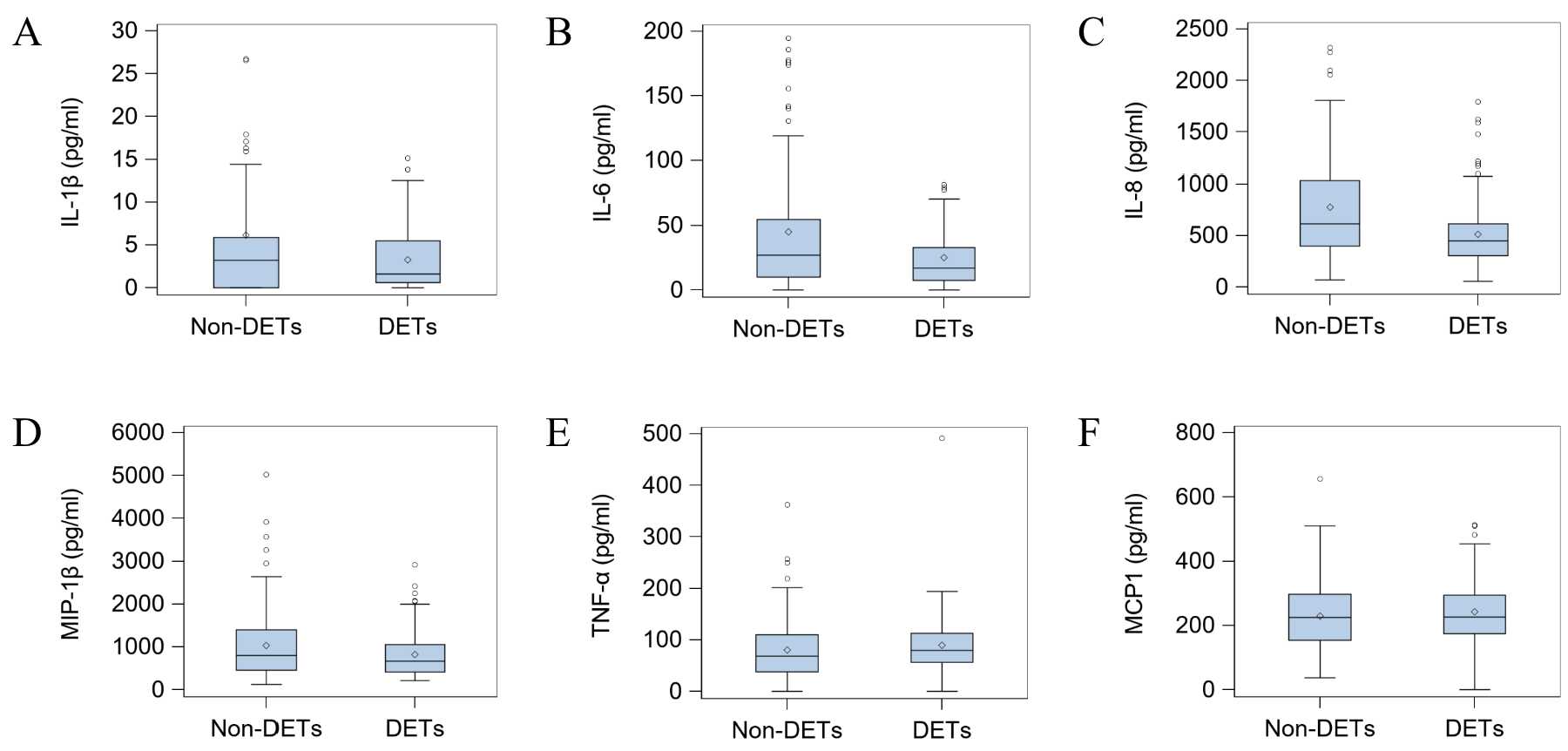


**Supplemental Figure 1.** **Comparison of serum pro-inflammatory cytokines and chemokines between 125 DETs and 126 non-DETs.** (A) IL-1β（*P* = 0.4684). (B) IL-6（*P* = 0.0017). (C) IL-8（*P* < 0.0001). (D) MIP-1β（*P* = 0.1176). (E) TNF-α（*P* = 0.0594). (F) MCP1（*P* = 0.2468). Wilcoxon signed-rank test was used to compare values from both groups.

**Supplemental Table 1. Principal component analysis of 7 biosensor genes in 124 DETs and 123 non-DETs (delta Ct)**

| Variable | PC1 | PC2 | PC3 | PC4 | PC5 | PC6 | PC7 |
| --- | --- | --- | --- | --- | --- | --- | --- |
| CCL2 | **0.37** | -0.30 | 0.38 | 0.68 | 0.04 | -0.09 | -.40 |
| CCL5 | **0.41** | -0.29 | 0.36 | -0.27 | -0.26 | 0.61 | 0.33 |
| CXCL8 | **0.33** | -0.04 | -0.77 | 0.36 | 0.15 | 0.35 | 0.15 |
| CXCL12 | **0.38** | **0.44** | -0.10 | 0.08 | -0.75 | -0.29 | 0.04 |
| ICAM | **0.45** | 0.03 | -0.16 | -0.55 | 0.17 | 0.004 | -0.67 |
| SELP | 0.19 | **0.77** | 0.31 | 0.13 | 0.44 | 0.24 | 0.10 |
| VCAM | **0.46** | -0.18 | 0.03 | -0.15 | 0.36 | -0.60 | 0.50 |
| Total variance explained | 0.39 | 0.16 | 0.13 | 0.10 | 0.09 | 0.08 | 0.06 |

**Supplemental Table 2. Principal component analysis of blood cytokine and chemokine in 125 DETs and 126 non-DETs**

| Serum marker | Prin1 | Prin2 | Prin3 | Prin4 | Prin5 | Prin6 | Prin7 |
| --- | --- | --- | --- | --- | --- | --- | --- |
| IL-6 (pg/ml) | **0.51** | 0.03 | -.22 | 0.11 | 0.46 | -0.27 | -0.62 |
| TNF (pg/ml) | **0.50** | -0.09 | 0.04 | 0.10 | -0.26 | 0.80 | -0.14 |
| IL-8 (pg/ml) | **0.51** | -0.12 | -0.38 | -0.04 | 0.18 | -0.12 | 0.73 |
| MIP-1β (pg/ml) | **0.37** | **0.47** | 0.13 | 0.12 | -0.66 | -0.42 | -0.006 |
| IL-1β (pg/ml) | 0.09 | **0.60** | 0.15 | -0.71 | 0.26 | 0.18 | 0.04 |
| MCP1 (pg/ml) | -0.15 | **0.60** | -0.03 | 0.66 | 0.32 | 0.21 | 0.17 |
| CRP (mg/L) | 0.25 | -0.19 | **0.87** | 0.13 | 0.28 | -0.12 | 0.17 |
| Total variance explained | 0.368 | 0.200 | 0.134 | 0.114 | 0.077 | 0.065 | 0.043 |

**Supplemental Table 3. Pattern of triangles between diesel exhaust exposure, serum inflammation, and biosensor responses in 117 DETs and 122 non-DETs**

| Mediator | Pattern | Diesel mediator association | | |  | Mediator biosensor association | | |  | Diesel biosensor association | | |
| --- | --- | --- | --- | --- | --- | --- | --- | --- | --- | --- | --- | --- |
|  |  | Estimate (a) | StdErr | Raw P |  | Estimate (b) | StdErr | Raw P |  | Estimate (c') | StdErr | Raw P |
| Serum inflammation PC1 | Null | -0.2441 | 0.1380 | 0.0782 |  | -0.0910 | 0.0587 | 0.1223 |  | 0.2334 | 0.1244 | 0.0619 |
| Serum inflammation PC2 | Null | -0.1299 | 0.0950 | 0.1728 |  | -0.0260 | 0.0855 | 0.7617 |  | 0.2505 | 0.1249 | 0.0460 |
| Serum inflammation PC3 | Null | 0.1163 | 0.0852 | 0.1736 |  | -0.1536 | 0.0948 | 0.1064 |  | 0.2707 | 0.1241 | 0.0301 |

Note: The estimate for internal dose of diesel exhaust exposure for affecting biosensor PC was 0.2541 (c) with 0.1241 as the standard deviation. *P* for association was 0.0417.

**Supplemental Table 4. Pattern of triangles between diesel exhaust exposure, plasma metabolites and biosensor responses in 124 DETs and 123 non-DETs**

| Metabolite | Pattern | Diesel metabolite asso | | | |  | Metabolite biosensor asso | | |  | Diesel biosensor asso | | |
| --- | --- | --- | --- | --- | --- | --- | --- | --- | --- | --- | --- | --- | --- |
|  |  | Estimate (a) | StdErr | Raw P | FDR |  | Estimate (b) | StdErr | Raw P |  | Estimate (c') | StdErr | Raw P |
| Fumarate | I | -0.0015 | 0.0209 | 0.9446 | 0.9591 |  | 1.0353 | 0.3593 | 0.0043 |  | 0.2637 | 0.1159 | 0.0238 |
| Nicotinic-acid | I | -0.0150 | 0.0497 | 0.7624 | 0.8456 |  | -0.4221 | 0.1504 | 0.0054 |  | 0.2568 | 0.1161 | 0.0279 |
| N-methylarginine | S | -0.0518 | 0.0242 | 0.0333 | 0.1021 |  | 0.8503 | 0.3111 | 0.0067 |  | 0.3138 | 0.1174 | 0.0080 |
| cGMP | M | -0.0919 | 0.0297 | 0.0022 | 0.0172 |  | -0.6693 | 0.2507 | 0.0081 |  | 0.2035 | 0.1185 | 0.0873 |
| Xanthurenic-acid | I | -0.0011 | 0.0388 | 0.9766 | 0.9841 |  | -0.4311 | 0.1930 | 0.0264 |  | 0.2668 | 0.1167 | 0.0231 |
| Homoserine | I | 0.0272 | 0.0197 | 0.1698 | 0.3502 |  | -0.7781 | 0.3800 | 0.0417 |  | 0.2877 | 0.1174 | 0.0150 |

Note: Internal dose of diesel exhaust exposure was defined as the PC1 extracted from six urinary metabolites which has shown an excellent association with diesel exhaust exposure status. This PC was converted into an ordered categorical variable with three values (0, 1, 2) with each group having same number of subjects to calculate the estimate for the dose-response. Association (a) between internal dose of diesel exhaust exposure and natural-log transformed serum metabolite levels was assessed using generalized linear model with adjustment for age, obesity, and internal dose for cigarette smoking. Generalized linear model was used to assess association (c') between internal dose of diesel exhaust exposure and biosensor PC with age, obesity, internal of for cigarette smoking, passage of cells, and natural-log transformed serum metabolite levels (b) as covariate for adjustment. Generalized linear model was used to assess the association between internal dose of diesel exhaust exposure and biosensor PC in 124 DETs and 123 non-DETs with adjustment for age, obesity, internal of for cigarette smoking, and passage of cells. The estimate for internal dose of diesel exhaust exposure was 0.2659 (c) with 0.1177 as the standard deviation. P for association was 0.0247.

**Supplemental Table 5. Expression of apoptosis-related genes in *ex vivo* biosensor cells treated with the plasma of diesel exhaust exposure workers**

| Gene | FPKM.NC | FPKM.DEP | logFC | P.Value |
| --- | --- | --- | --- | --- |
| GADD45B | 30.5330796 | 43.94581978 | 0.564347046 | 0.029026968 |
| ATF3 | 17.38037902 | 24.5782286 | 0.532563353 | 0.005321821 |
| EREG | 93.50598053 | 130.6058464 | 0.521993885 | 0.008035902 |
| JUN | 31.96073896 | 44.05926085 | 0.503735911 | 9.67E-08 |
| TNFRSF12A | 1099.495108 | 1501.866647 | 0.492052001 | 0.000147616 |
| IGFBP6 | 29.44072719 | 39.24309029 | 0.45247209 | 0.002032014 |
| GADD45A | 28.03667723 | 36.02051063 | 0.403970923 | 0.000628308 |
| BMP2 | 49.73542962 | 63.63530878 | 0.393405893 | 0.001828039 |
| MGMT | 7.156959469 | 9.056865375 | 0.38288931 | 0.000597521 |
| GPX4 | 54.96125635 | 69.46596184 | 0.379130118 | 0.000150093 |
| GPX1 | 284.9098383 | 356.3790885 | 0.366279044 | 0.001727388 |
| SMAD7 | 5.59011015 | 6.888479267 | 0.340423995 | 0.004832157 |
| TSPO | 68.60852178 | 83.86565775 | 0.333611504 | 0.001686076 |
| IFITM3 | 411.151831 | 496.4977971 | 0.315704564 | 0.00142374 |
| RHOT2 | 583.3485192 | 702.4481167 | 0.310391461 | 0.000472147 |
| BIK | 11.30192506 | 13.55737828 | 0.302760986 | 0.005883654 |
| TAP1 | 30.82884582 | 36.782298 | 0.294047898 | 0.013383702 |
| FDXR | 71.69908718 | 85.20182339 | 0.291190007 | 0.013006752 |
| RHOB | 231.7315664 | 274.6403017 | 0.287727377 | 0.000314601 |
| PPP2R5B | 86.4256712 | 102.5626437 | 0.287070749 | 0.004181429 |
| ENO2 | 145.9654815 | 173.2357229 | 0.283785399 | 0.044461866 |
| GPX3 | 308.698645 | 364.7472891 | 0.282953943 | 0.00228528 |
| IL1A | 518.590293 | 607.108772 | 0.267953476 | 0.039596368 |
| TIMP3 | 226.4472699 | 262.6355294 | 0.254874461 | 0.032224511 |
| PMAIP1 | 102.220047 | 118.4202811 | 0.248444218 | 0.045621585 |
| GSR | 289.9423561 | 331.2664758 | 0.232219224 | 0.013146997 |
| HMGB2 | 45.3614725 | 51.13635869 | 0.21140336 | 0.039236004 |
| BAX | 19.2935039 | 21.64485893 | 0.210302655 | 0.034948733 |
| ERBB2 | 1399.710139 | 1558.170663 | 0.195783825 | 0.008241537 |
| LMNA | 660.1510278 | 729.008405 | 0.184567701 | 0.04446993 |
| BNIP3L | 458.4783299 | 388.7925582 | -0.195902795 | 0.013627282 |
| CD44 | 54.55983143 | 46.16209024 | -0.19925298 | 0.017970775 |
| RNASEL | 127.5495061 | 107.7854741 | -0.202548233 | 0.04898546 |
| CASP8 | 53.90998075 | 44.14248813 | -0.24377542 | 0.014932442 |
| DPYD | 2.221496981 | 1.814362139 | -0.245859196 | 0.022489059 |
| CYLD | 42.43560184 | 34.71502541 | -0.249514195 | 0.004960826 |
| SATB1 | 51.97567686 | 38.79418018 | -0.381405075 | 0.000455398 |
| BRCA1 | 2.463284425 | 1.715342533 | -0.472161095 | 0.000260523 |

Note: The *ex vivo* biosensor assay was conducted by using primary human bronchial epithelial cells. The cleaved RNA fragments were then reverse transcribed using SuperScriptTM II Reverse Transcriptase (Invitrogen, USA) to generate cDNA. We then followed the vendor's recommended protocol for 2*150 bp paired-end sequencing (PE150) on an Illumina NovaseqTM 6000 platform. The differentially expressed genes (DEGs) between the DEE exposure and control groups were calculated using the edgeR packet (version 3.32.1) of R software 4.0.4. The apoptosis-related genes in the DEGs were extracted to describe the effect of diesel exhaust exposure on apoptosis.
